# Large language models and child mortality: opportunities and challenges in answering public queries on under-5 causes

**DOI:** 10.3389/fpubh.2026.1646475

**Published:** 2026-05-05

**Authors:** Yi Yang, Tingxi Zhu, Hongju Chen, Yao Zhang, Chao Zhang, Dengjun Liu, Yanxia Mao, Jiaxi Wu, Tao Xiong

**Affiliations:** 1 Department of Pediatrics, West China Second University Hospital, Sichuan University, Chengdu, China; 2 Key Laboratory of Birth Defects and Related Diseases of Women and Children, Ministry of Education, Sichuan University, Chengdu, China; 3 Department of Pediatric Otolaryngology Head and Neck Surgery, West China Second University Hospital, Sichuan University, Chengdu, China; 4 West China Biomedical Big Data Center, West China Hospital, Sichuan University, Chengdu, China; 5 Med-X Center for Informatics, Sichuan University, Chengdu, China; 6 Children's Medicine Key Laboratory of Sichuan Province, Chengdu, China

**Keywords:** accuracy, actionability, child mortality, comprehensiveness, large language models, pediatrics, public health communication, quality

## Abstract

**Background:**

Reducing under-5 mortality remains a global health priority. Large language models (LLMs) are increasingly used by the public to access medical information. However, current evidence evaluating LLMs’ performance in public-facing child health communication is scarce.

**Methods:**

We selected the top five search terms related to each of the five leading causes of under-5 mortality (prematurity, pneumonia, birth asphyxia, malaria, and diarrhoea) using Google Trends, generating 25 representative public queries. Responses were collected from four LLMs (ChatGPT-4.0, Claude 3.5 Sonnet, Bing AI, and Gemini) and independently evaluated by four pediatricians. We used the DISCERN instrument for information reliability; 5-point Likert scales for accuracy, completeness, and comprehensibility; Flesch Reading Ease (FRE) and Flesch–Kincaid Grade Level (FKGL) indices for readability; and the Patient Education Materials Assessment Tool for Printable Materials (PEMAT-P) for understandability and actionability. Differences among models were evaluated with Kruskal–Wallis and ANOVA tests, with statistical significance set at *p* < 0.05.

**Results:**

We found significant performance variations among the four models across most evaluation metrics. Bing AI achieved the highest total DISCERN score (median 42) and the highest reliability subscore (Section A median 28). Claude consistently underperformed across multiple domains. Notably, readability was poor for all models, with high language complexity (mean FKGL score 12.4). Critically, actionability scores were near zero for all models on the PEMAT-P scale, reflecting a universal lack of clear and practical behavioral guidance.

**Conclusion:**

While LLMs can generally provide accurate health information, limitations in readability and actionability restrict their practical application in public health communication. Future development should prioritize language simplification and clearer behavioral guidance to enhance their value in public-facing child health communication.

## Introduction

Reducing under-5 mortality remains a global priority. Over the past three decades, more than 257 million children under 5 years have died globally. Annual deaths have declined over time and are estimated at approximately 5 million per year in recent years, with most deaths attributable to preventable or treatable conditions (1). According to the 2023 report by the United Nations Inter-agency Group for Child Mortality Estimation, the five leading causes of neonatal and under-5 mortality are prematurity (18%), pneumonia (14%), birth asphyxia (12%), malaria (9%), and diarrhoea (9%) (2). Against this backdrop, the internet has become an increasingly important source of health information. Access to online resources allows caregivers to better understand disease, supports early intervention, and may help reduce anxiety (3).

In recent years, natural language processing technologies based on deep learning have advanced rapidly. Large language models (LLMs), with their convenience and robust interactive capabilities, are becoming essential tools for public access to medical information (4). Trained on massive collections of text from articles, websites, and other open sources, LLMs are capable of understanding and generating sophisticated language (5). Prominent examples include OpenAI’s ChatGPT-4.0, recognized for its reasoning and text generation capabilities (6); Claude 3.5 Sonnet by Anthropic, designed with a focus on safety and controllability (7); Microsoft’s Bing AI (also known as Microsoft Copilot), tailored for intelligent productivity applications (8); and Google’s Gemini, launched in 2024 as an evolution of Bard, noted for its strengths in search and information synthesis (9). These models are increasingly used in health consultation, decision support, and medical content generation.

In recent years, LLMs have attracted growing attention in pediatric clinical research (10–14). Early studies have explored their ability to predict acute asthma exacerbations in children (10), support child health management (12), diagnostic reasoning accuracy in pediatric case analysis (13), and aid decision-making in pediatric emergency care (14). These studies highlight the potential of LLMs to enhance diagnostic efficiency and quality in clinical pediatrics. However, most existing research focuses on LLMs as tools for healthcare professionals. Notably, far less attention has been paid to another major user group—the general public—who increasingly turn to LLMs for pediatric health information. Given the substantial impact of LLM-generated public-facing health information on decision-making, it is crucial to systematically evaluate the performance of LLMs in responding to pediatric health inquiries.

As online queries related to childhood diseases continue to rise annually (15), the influence of LLM-generated content on parental health literacy and care decisions is potentially profound (16). Despite this growing significance, research systematically evaluating public-facing LLM responses in pediatric health information remains scarce. To address this critical gap, we evaluated the responses of four widely used LLMs—ChatGPT-4.0, Claude 3.5 Sonnet, Bing AI, and Gemini—in responding to commonly searched questions about five major causes of childhood mortality: prematurity, pneumonia, birth asphyxia, malaria, and diarrhoea. We examined the responses in terms of their quality, accuracy, comprehensiveness, readability, and actionability.

## Methods

### Search term selection and LLMs configuration

Google Trends provides data on the popularity of search terms on the Google search engine over time (17). Using this tool, we identified the top five most frequently searched terms worldwide from August 1, 2023 to August 1, 2024 related to prematurity, pneumonia, birth asphyxia, malaria, and diarrhoea. A total of 25 representative search queries were generated (see Table 1).

**Table 1:** Search terms derived from Google Trends related to five high-mortality pediatric diseases.

Prematurity	Pneumonia	Birth asphyxia	Malaria	Diarrhoea
What is retinopathy prematurity	Pneumonia symptoms among newborns and children younger than 5	Birth asphyxia meaning	Malaria symptoms among newborns and children younger than 5	What is diarrhoea among newborns and children younger than 5
When is world prematurity day	What is pneumonia among newborns and children younger than 5	What is birth asphyxia	What is malaria among newborns and children younger than 5	Diarrhoea meaning among newborns and children younger than 5
Prematurity icd 10	Pneumonia contagious among newborns and children younger than 5	Birth asphyxia definition	What is dengue among newborns and children younger than 5	Diarrhoea symptoms among newborns and children younger than 5
Apnea of prematurity	Walking pneumonia among newborns and children younger than 5	Birth asphyxia causes	Malaria treatment among newborns and children younger than 5	Causes diarrhoea among newborns and children younger than 5
What is prematurity	Pneumonia treatment among newborns and children younger than 5	What is neonatal	Malaria test among newborns and children younger than 5	Sickness and diarrhoea among newborns and children younger than 5

The 25 selected queries were independently entered into four LLMs: ChatGPT (paid version 4.0), Claude (free version 3.5 Sonnet), Bing AI (configured in “balanced mode”), and Gemini (with default settings), using the most recent publicly accessible versions available as of September 10, 2024. To minimize potential bias from dialogue memory and prior user-specific personalization, each question was submitted in a separate session using newly registered accounts without recorded individual usage history, thereby ensuring isolated outputs. All responses were converted into plain text and anonymized by removing model-identifying elements. These were then evaluated in a blinded fashion by four pediatricians. The four evaluators were all pediatricians with different levels of seniority, including two attending pediatricians (YY and YZ), one associate senior pediatrician (HJC), and one senior pediatrician (TX). All assessments were performed independently using standardized evaluation instruments.

### Evaluation tools

The responses generated by LLMs were evaluated using the following standardized tools: the DISCERN instrument to assess the reliability of information (18); Likert scales to rate accuracy, completeness, and understandability (19); the Flesch Reading Ease (FRE) and Flesch–Kincaid Grade Level (FKGL) indices to measure readability (20); and the Patient Education Materials Assessment Tool for Printable Materials (PEMAT-P) to assess understandability and actionability (21).

### Readability (FRE and FKGL)

Readability reflects how easily a text can be understood by its target audience, and depends on various elements such as sentence construction, vocabulary length, and linguistic complexity (22). In this study, we evaluated the readability of LLM-generated responses using two established metrics: FRE and FKGL. FRE scores range from 0 (difficult) to 100 (easy), with scores below 30 considered challenging for general readers (20). FKGL scores indicate U.S. school grade levels appropriate for understanding the text (23). FKGL scores range from 5 (easy) to 16 (difficult) (24), and health education guidelines typically recommend that materials be written at below an eighth-grade reading level to ensure comprehensibility for the general population (25). FRE and FKGL scores were automatically calculated using Microsoft Office Word 2023, ensuring standardization and objectivity in the assessment process.

### Reliability and accuracy (DISCERN)

The DISCERN instrument is a validated tool designed to assess the reliability and quality of treatment information intended for the general public. It helps determine whether a publication can serve as a trustworthy reference for treatment decisions, whether information sources are clearly cited, and whether the content contains common forms of misinformation or inaccuracies. The tool is simple to use and can be independently applied by non-experts without requiring external resources or specialist input (18). DISCERN includes 16 items across three sections: general reliability (Section A, questions 1–8), quality of information on treatment choices (Section B, questions 9–15), and an overall quality rating (Section C, question 16). Each item is rated on a scale from 1 (poor) to 5 (excellent), providing a robust assessment of information reliability (26, 27). Full details of the items and scoring criteria are listed in Supplementary Table S1.

### Accuracy, completeness, and comprehensibility (Likert scales)

The Likert scale is a widely applied tool in social sciences for structured evaluation, offering a simple and quantifiable method to assess attitudes toward specific statements (28). In this study, the accuracy, completeness, and comprehensibility of responses were rated using adapted 5-point Likert scales (19), ranging from 1 (poor) to 5 (excellent) (23). Scoring criteria were developed with reference to established medical knowledge and the logical coherence of the content, aiming to ensure both the professional reliability of the assessments and their relevance to public comprehension. Detailed scoring standards are provided in Supplementary Table S2.

### Understandability and actionability (PEMAT-P)

PEMAT-P was used to evaluate the understandability and actionability of health information. This tool is particularly suited for assessing materials across diverse levels of health literacy and social backgrounds (21). PEMAT-P includes 24 items in total: 17 items assess understandability (items 1–12 and 15–19), and 7 items assess actionability (items 20–26). For each item, evaluators select “agree” or “disagree.” Final scores are calculated as the proportion of agreed items out of applicable items, yielding a percentage score. Higher percentages indicate greater public comprehension and actionable guidance (21). Detailed item descriptions are provided in Supplementary Table S3.

### Statistical analysis

The Shapiro–Wilk test was used to assess the normality of data distribution. Continuous variables with a normal distribution were presented as mean ± SD and compared between groups using analysis of variance (ANOVA). Non-normally distributed continuous variables were presented as median (minimum-maximum) and compared using the Kruskal–Wallis rank-sum test. A *p-*value <0.05 was considered statistically significant. All statistical analyses were performed using SPSS version 26 (IBM SPSS Statistics for Windows, Armonk, NY, USA).

## Results

Four pediatricians independently evaluated the answers generated by ChatGPT-4, Claude 3.5 Sonnet, Bing AI, and Gemini to the five most common public queries concerning each of the 5 leading causes of pediatric mortality: prematurity, pneumonia, birth asphyxia, malaria, and diarrhoea. In total, 100 responses were collected, and each pediatrician assessed all responses, yielding 400 evaluation records.

Before formal analysis, inter-rater consistency among the four pediatricians was tested. No statistically significant differences were observed among evaluators (see Supplementary Table S4), indicating acceptable scoring consistency.

### Readability (FRE and FKGL)

**Figure 1:**
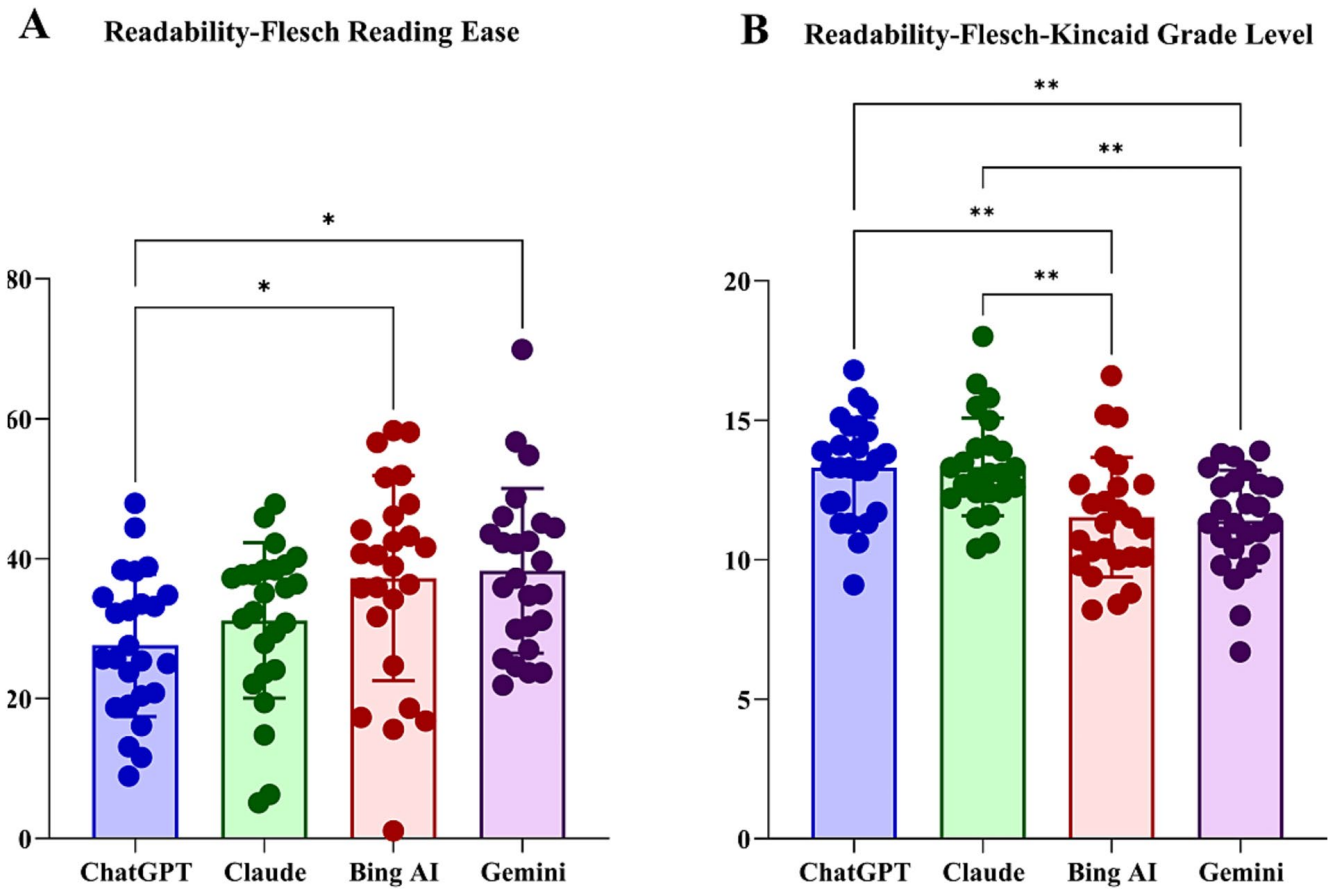
Readability comparison of large language model responses using Flesch Reading Ease **(A)** and Flesch–Kincaid Grade Level **(B)** scores. *p < 0.05; **p < 0.01 for *post-hoc* comparisons.

The overall FRE score across all models was 33.56 ± 12.65, indicating generally poor readability. Significant differences were noted among the models (*F* = 4.38, *p* = 0.006; Table 2). ChatGPT responses were significantly less readable compared with those from Gemini and Bing AI [mean 27.62 vs. 37.19 and 38.25, respectively; adjusted (adj) *p* < 0.05; Figure 1A].

**Table 2:** Readability scores of four large language models based on Flesch Reading Ease and Flesch–Kincaid Grade Level.

Readability (mean ± SD)	FRE	FKGL
ChatGPT	27.62 ± 10.19	13.30 ± 1.79
Claude	31.17 ± 11.11	13.33 ± 1.75
Bing AI	37.19 ± 14.66	11.52 ± 2.15
Gemini	38.25 ± 11.77	11.40 ± 1.81
Total	33.56 ± 12.65	12.39 ± 2.07
*F*	4.38	8.11
*p*	**0.006**	**<0.001**

FRE, Flesch Reading Ease; FKGL, Flesch–Kincaid Grade Level.

Bold values indicate statistical significance (*p* < 0.05).

The overall FKGL score was 12.39 ± 2.07, indicating high linguistic complexity, with significant model differences (*F* = 8.11, *p* < 0.001; Table 2). Specifically, ChatGPT and Claude produced significantly more complex text (mean FKGL 13.3 and 13.33, respectively) compared with Bing AI and Gemini (mean FKGL 11.52 and 11.40, respectively; adj *p* < 0.05; Figure 1B).

### Reliability and accuracy (DISCERN)

**Figure 2:**
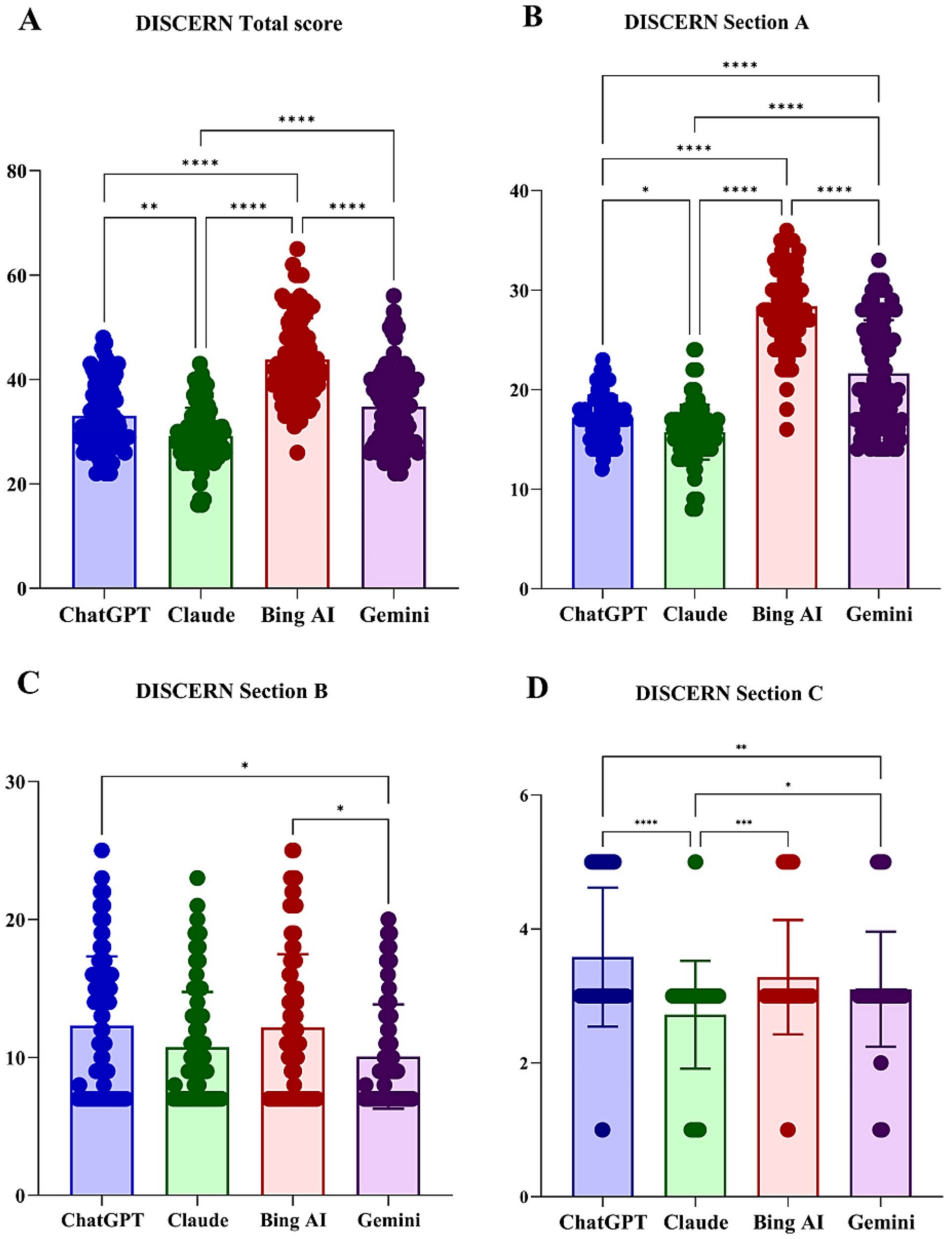
Assessment of information reliability and accuracy using DISCERN scores across large language models: **(A)** total DISCERN score; **(B)** Section A, reliability; **(C)** Section B, quality of information on treatment choices; and **(D)** Section C, overall quality rating.*p < 0.05; **p < 0.01; ***p < 0.001; ****p < 0.0001 for Bonferroni-adjusted pairwise comparisons.

Significant differences among models were observed in both the total DISCERN score and across all sections A–C (*p* < 0.01) (Table 3). The overall median DISCERN score was 34 (interquartile range [IQR] 28–41), indicating generally high reliability of treatment-related information. Bing AI achieved the highest total score (median 42), while Claude had the lowest (median 29) (*H* = 144.62, *p* < 0.001; Figure 2A). In Section A (reliability), Bing AI achieved the highest median score (28) and Claude the lowest (15) (*H* = 228.32, *p* < 0.001; Figure 2B). In Section B (details of treatment options), Gemini scored slightly lower than ChatGPT and Bing AI (*H* = 12.2, *p* = 0.007; Figure 2C). In Section C (overall judgment), ChatGPT scored significantly higher than Claude and Gemini (*H* = 43.53, *p* < 0.001; Figure 2D).

**Table 3:** Comparative DISCERN scores across four large language models.

DISCERN M (*Q*₁, *Q*₃)	Section A	Section B	Section C	Total score
ChatGPT	17 (16, 19)	11.5 (7, 16)	3 (3, 5)	32 (28, 38)
Claude	15 (14, 17)	10 (7, 13)	3 (3, 3)	29 (26, 32)
Bing AI	28 (26, 32)	11 (7, 15)	3 (3, 3)	42 (38, 50)
Gemini	20.5 (17, 26)	9 (7, 12)	3 (3, 3)	35 (28, 40)
Overall	18 (16, 26)	10 (7, 14)	3 (3, 3)	34 (28, 41)
H	228.32	12.2	43.53	144.62
*p*	**<0.001**	**0.007**	**<0.001**	**<0.001**

M, median; Q₁, 1st quartile; Q₃, 3rd quartile.

Bold values indicate statistical significance (*p* < 0.05).

### Accuracy, completeness, and comprehensibility (Likert scales)

**Figure 3:**
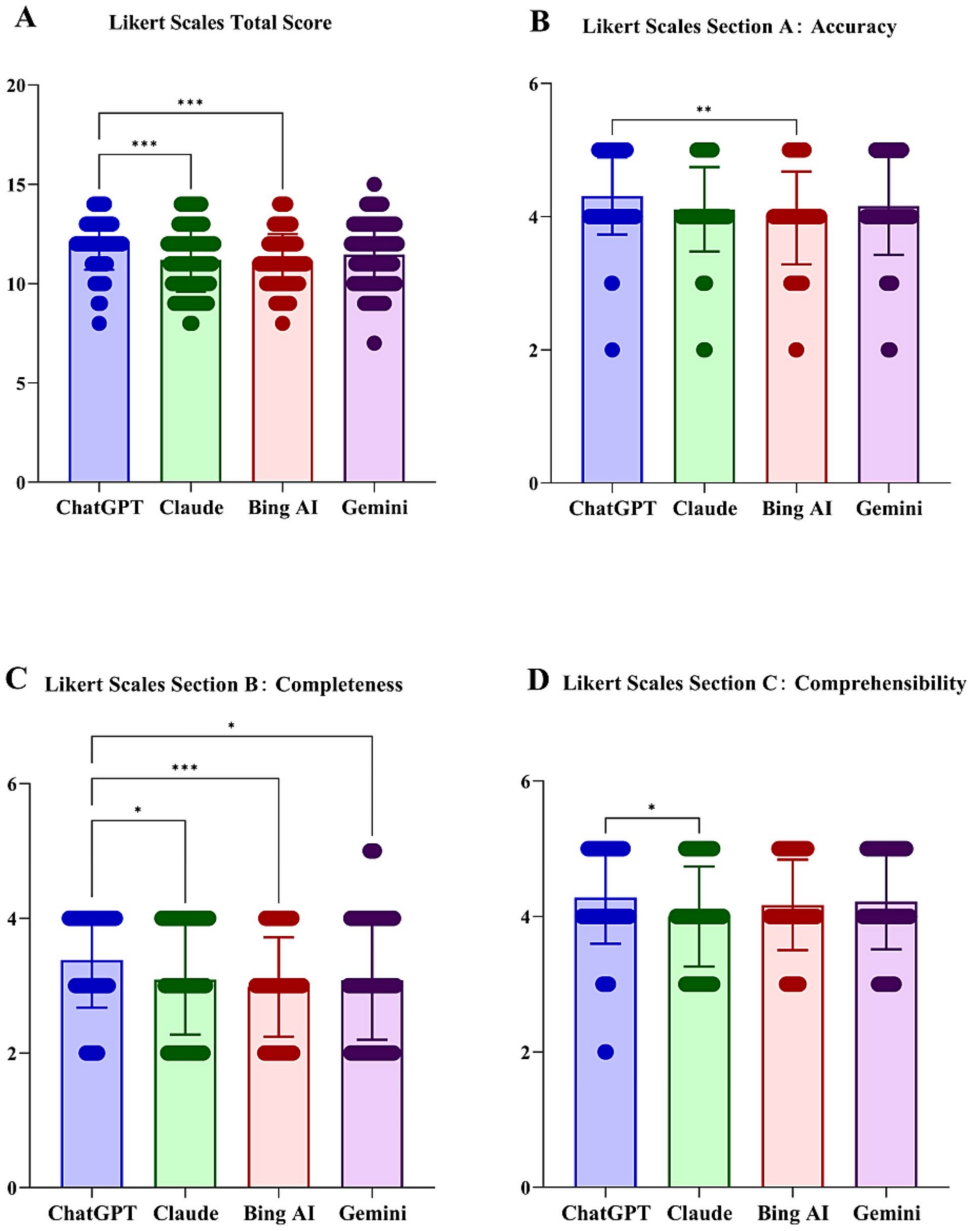
Likert scale comparison for accuracy, completeness, and comprehensibility: **(A)** total Likert score; **(B)** Section A, accuracy; **(C)** Section B, completeness; and **(D)** Section C, comprehensibility. *p < 0.05; **p < 0.01; ***p < 0.001 for Bonferroni-adjusted pairwise comparisons.

Likert scale evaluations revealed significant differences not only in the total score but also across all three assessment dimensions (*p* < 0.05; Table 4). The overall median total score was 12 (IQR 10–13), with ChatGPT and Gemini scoring higher than Claude and Bing AI (*H* = 21.93, *p* < 0.001; Figure 3A). For Section A (accuracy), the median score across all models was 4 (IQR 4–5), particularly between ChatGPT and Claude (adj *p* < 0.05; Figure 3B). Section B (completeness) had an overall median score of 3 (IQR 2–4), with ChatGPT performing significantly better than the other models (median 4 vs. 3; adj *p* < 0.05; Figure 3C). In Section C (comprehensibility), the median score was 4 (IQR 4-5) with minor but significant differences (*H* = 8.99, *p* = 0.029), mainly between ChatGPT and Claude or Bing AI (Figure 3D).

**Table 4:** Likert scale evaluation of accuracy, completeness, and comprehensibility of responses from four large language models.

Likert scales M (*Q*₁, *Q*₃)	Section A: accuracy	Section B: completeness	Section C: comprehensibility	Total score
ChatGPT	4 (4, 5)	4 (3, 4)	4 (4, 5)	12 (11, 13)
Claude	4 (4, 4)	3 (2, 4)	4 (3, 5)	11 (10, 12)
Bing AI	4 (4, 4)	3 (2, 4)	4 (4, 5)	11 (10, 12)
Gemini	4 (4, 5)	3 (2, 4)	4 (4, 5)	12 (10, 13)
Overall	4 (4, 5)	3 (2, 4)	4 (4, 5)	12 (10, 13)
H	13.06	14.67	8.99	21.93
*p*	**0.005**	**0.002**	**0.029**	**<0.001**

M, median; Q₁, 1st quartile; Q₃, 3st quartile.

Bold values indicate statistical significance (*p* < 0·05).

### Understandability and actionability (PEMAT-P)

**Figure 4:**
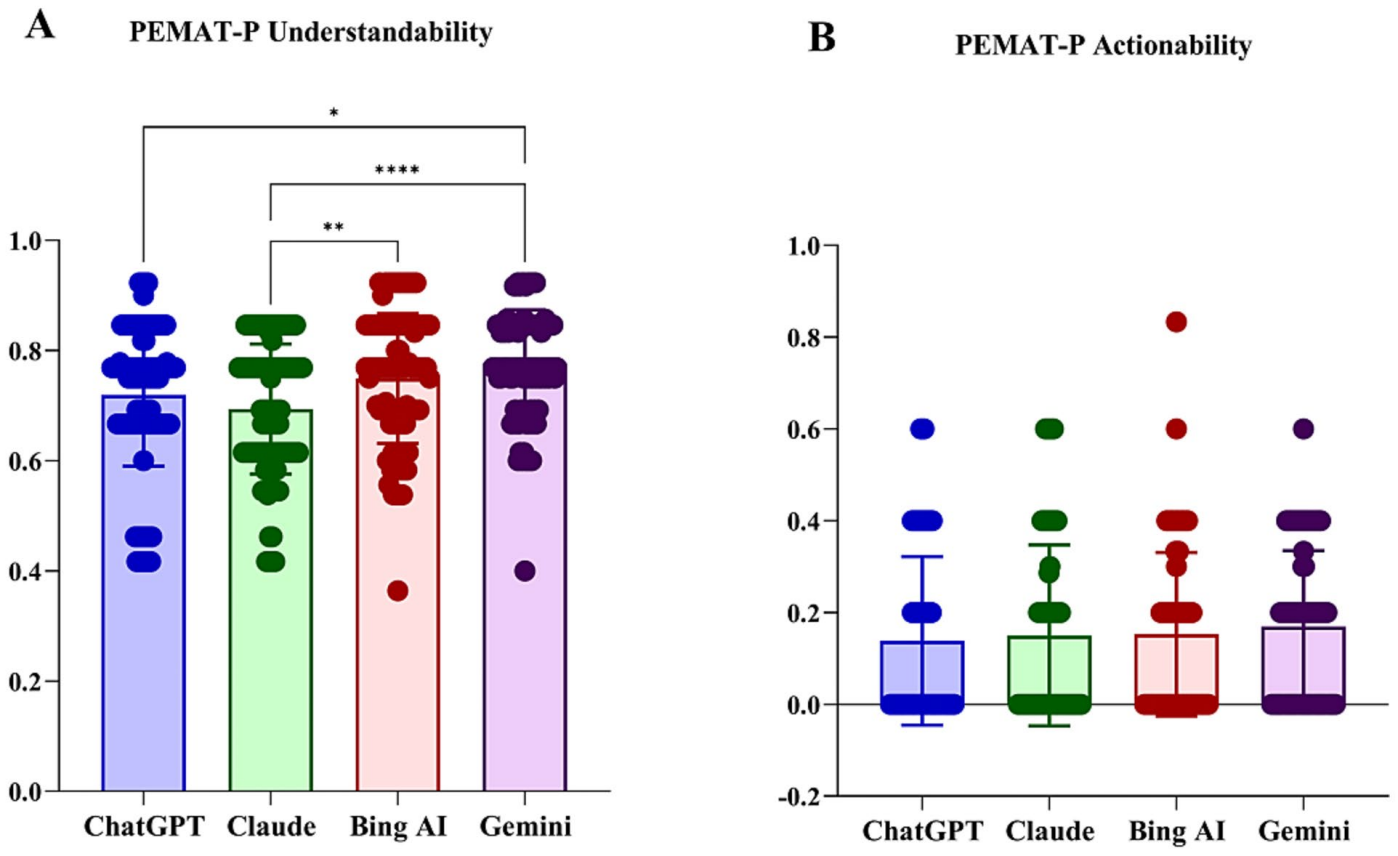
Understandability and actionability assessment by the Patient Education Materials Assessment Tool for Printable Materials scale: **(A)** understandability; and **(B)** actionability. *p < 0.05; **p < 0.01; ****p < 0.001 for Bonferroni-adjusted comparisons.

The PEMAT-P assessment showed significant differences in understandability across the four models (*H* = 23.39, *p* < 0.001; Table 5). Claude produced the least understandable outputs (median 0.69), scoring significantly lower than Gemini and Bing AI (median 0.77; adj *p* < 0.05; Figure 4A). In contrast, actionability scores were uniformly low across all models, with values close to zero and no statistically significant differences detected (*p* = 0.362; Figure 4B), suggesting that none of the LLMs provided clear or actionable behavioral guidance.

**Table 5:** Patient Education Materials Assessment Tool for Printable Materials assessment of understandability and actionability of model-generated content.

PEMAT-P M (*Q*₁, *Q*₃)	Understandability	Actionability
ChatGPT	0.75 (0.67, 0.78)	0 (0, 0.2)
Claude	0.69 (0.62, 0.77)	0 (0, 0.2)
Bing AI	0.77 (0.67, 0.85)	0.10 (0, 0.23)
Gemini	0.77 (0.75, 0.85)	0.2 (0, 0.31)
Overall	0.77 (0.67, 0.85)	0 (0, 0.2)
H	23.39	3.2
*p*	**<0.001**	0.362

PEMAT-P, Patient Education Materials Assessment Tool for Printable Materials.

M, median; Q₁, 1st quartile; Q₃, 3rd quartile.

Bold values indicate statistical significance (*p* < 0.05).

## Discussion

In recent years, research into the application of LLMs across various medical fields has advanced rapidly. However, the effectiveness of LLMs in supporting public access to pediatric health information remains inadequately evaluated. This study addresses this gap by systematically assessing, for the first time, the performance of four LLMs—ChatGPT, Claude, Bing AI, and Gemini—in responding to questions related to the leading causes of under-5 mortality, including prematurity, pneumonia, birth asphyxia and trauma, malaria, and diarrhoea.

Overall, the LLMs demonstrated good performance in the accuracy of medical information, but notable differences were observed across dimensions. Bing AI showed the best performance in citation of sources and structural rigor, ChatGPT achieved the highest scores in content completeness, and Gemini excelled in language understandability. Claude, however, consistently scored lower across multiple domains. These findings suggest that while LLMs have the potential to generate high-quality medical content, they do not yet meet the standards required for clinical use or health communication. Previous studies have also highlighted the limitations of LLMs in handling medical literature, applying specialized terminology, and integrating comprehensive information (29, 30). More critically, the issue of “hallucination”—the generation of plausible but factually incorrect information—remains a major safety concern (13, 31). Furthermore, the lack of individualized judgement capability suggests that, at present, LLMs are better suited as supplementary sources of medical information rather than as tools for clinical decision-making (32).

A major concern identified in our study was the consistently poor actionability across all models, reflected by near-zero PEMAT-P actionability scores. Responses rarely specified concrete user actions, provided step-by-step guidance, or included practical supports such as examples or tools, which are key components of actionability within the PEMAT-P framework. This pattern likely reflects a combination of factors. First, LLMs may adopt a risk-averse approach in medical contexts due to safety alignment, often defaulting to general recommendations rather than offering specific behavioral guidance. Second, current models may have limited capacity to translate medical knowledge into structured, actionable instructions for caregivers, particularly in breaking down tasks into clear, sequential steps. Finally, the opaque nature of LLM training data, including the uncertain quantity and quality of domain-specific medical content, may further constrain their ability to generate reliable and practical guidance. Together, these findings highlight a gap between information generation and real-world usability in health communication. This finding aligns with previous studies in oncology, ophthalmology, and urology, which similarly reported that LLM-generated health content frequently lacks explicit, practical recommendations (31, 33, 34). Our study highlights the limitation in actionable guidance and underscores the need for future model development to improve the clarity and actionability of health information, thereby enhancing the practical value of LLMs in public health communication.

Moreover, the linguistic complexity of responses across all evaluated LLMs was uniformly high (mean FKGL score 12.4), corresponding to readability suitable for university-level education rather than the general public. This issue is consistent with broader findings in health communication literature, where essential patient education materials commonly exceed recommended readability levels, thus limiting their practical utility and potentially adversely influencing healthcare decisions (35). Similar readability issues were reported by Robinson et al. (36), who noted that dermatologic information from ChatGPT and Bard contained excessive technical jargon, impeding public understanding. Our findings confirm that this problem also applies in the context of pediatric health communication and highlight the need for future LLMs development to prioritize simplified language and layperson-friendly expressions to improve readability and enhance accessibility for the general public.

This concern may be especially important in low- and middle-income countries (LMICs), where under-5 mortality remains highest and most children younger than 5 live in low- or lower-middle-income countries (37). Given the high FKGL observed in our study and the use of English-language queries, current LLM outputs may be difficult for many caregivers to use in settings where literacy barriers remain substantial (38). This challenge may be further compounded by language and infrastructure barriers, as many people in LMICs do not receive education in a language they speak and understand fluently, and under-resourced settings often face limited internet connectivity and unstable infrastructure (39, 40). Since current medical LLMs remain largely English-centred, they may risk widening digital health inequities unless future systems prioritize plain-language design, multilingual adaptation, and equitable deployment (41–43).

Building on our findings, we suggest several strategies to optimize the use of LLMs for public-facing pediatric health communication. First, integrating multi-modal formats such as text, images, and videos could enhance information visualization and improve understandability. Second, establishing a risk alert mechanism to notify users when models face uncertain or ambiguous queries could help guide users to seek professional medical advice. Thirdly, developing customized models specifically designed for pediatric health contexts, incorporating plain-language expression and actionable behavioral guidance, could better meet the needs of caregivers. Lastly, future research should also explore user feedback to evaluate the impact of model outputs on health literacy and behaviors, aiming to strike a balance between accuracy, readability, and utility. Such advancements will require ongoing collaboration between medical professionals, health communication experts, and AI developers.

This study has several limitations. It assessed LLM performance on a limited range of pediatric diseases, and the evaluation reflected model versions available at a specific time. In addition, the relatively small numbers of queries and evaluators may limit the breadth and generalizability of the findings, although inter-rater consistency analysis indicated acceptable agreement. Furthermore, the standardized single-turn design may not capture variation related to prompt phrasing or multi-turn interaction, and the use of English-language queries and responses may limit the broader applicability of the findings. Future research should expand the scope of diseases and queries evaluated, include more diverse linguistic settings, examine model performance under different prompting strategies, and longitudinally assess the real-world effectiveness of LLM-generated health information.

## Conclusion

This study compared the performance of four large language models in responding to public queries concerning high-mortality diseases in children under 5 years. Although the overall quality of information was satisfactory, deficiencies in readability and actionability were observed, limiting their practical utility for public use. Future efforts should focus on simplifying language, embedding clear behavioral guidance, and incorporating user feedback to better align model outputs with public needs and enhance their value in pediatric health communication.

## Data Availability

The original contributions presented in the study are included in the article/Supplementary material; further inquiries can be directed to the corresponding author.
