# Supplementary material for: Large language models and child mortality: opportunities and challenges in answering public queries on under-5 causes
Source: Front Public Health. 2026 May 5;14:1646475. doi: 10.3389/fpubh.2026.1646475 (PMC13183653; doi:10.3389/fpubh.2026.1646475)
Supplement: Supplementary file 1 [file Table_1.docx]

**Supplemental table 1: Full content of the DISCERN instrument**

| **Section A: Is the publication reliable?** | |
| --- | --- |
| 1 | Are the aims clear? |
| 2 | Does it achieve its aims? |
| 3 | Is it relevant? |
| 4 | Is it clear what sources of information were used to compile the publication (other than the author or producer)? |
| 5 | Is it clear when the information used or reported in the publication was produced? |
| 6 | Is it balanced and unbiased? |
| 7 | Does it provide details of additional sources of support and information? |
| 8 | Does it refer to areas of uncertainty? |
| **Section B: How good is the quality of information on treatment choices?** | |
| 9 | Does it describe how each treatment works? |
| 10 | Does it describe the benefits of each treatment? |
| 11 | Does it describe the risks of each treatment? |
| 12 | Does it describe what would happen if no treatment is used? |
| 13 | Does it describe how the treatment choices affect overall quality of life? |
| 14 | Is it clear that there may be more than one possible treatment choice? |
| 15 | Does it provide support for shared decision-making? |
| **Section C: Overall rating of the publication.** | |
| 16 | Based on the answers to all of the above questions, rate the overall quality of the publication as a source of information about treatment choices. |

**Supplemental table 2: Likert scale definitions**

| **Section A：Accuracy rating by a 5-point Likert scale** | |
| --- | --- |
| 1 | Completely incorrect. |
| 2 | More incorrect than correct. |
| 3 | Approximately equally correct and incorrect. |
| 4 | More correct than incorrect. |
| 5 | Correct. |
| **Section B：Completeness rating by a 5-point Likert scale** | |
| 1 | Incomplete, addresses some aspects of the question, but significant parts are missing or incomplete. |
| 2 | Not quite complete, with some important parts explained, but still not fully complete. |
| 3 | Adequate, addresses all aspects of the question and provides the minimum amount of information required to be considered complete. |
| 4 | Basically complete, with sufficient explanation, especially for the important parts. |
| 5 | Comprehensive, addresses all aspects of the question and provides additional information or context beyond what was expected. |
| **Section C：Comprehensibility rating by a 5-point Likert scale** | |
| 1 | Difficult to understand. |
| 2 | Most of it is difficult to understand. |
| 3 | Partly difficult to understand. |
| 4 | A small part of it is difficult to understand. |
| 5 | Easy to understand. |

**Supplemental table 3: Patient Education Materials Assessment Tool for Printable Materials (PEMAT-P) item list**

| **[Understandability](http://www.ahrq.gov/professionals/prevention-chronic-care/improve/self-mgmt/pemat/)** | |
| --- | --- |
| **Content** | |
| 1 | The material makes its purpose completely evident. |
| 2 | The material does not include information or content that distracts from its purpose. |
| **Word Choice & Style** | |
| 3 | The material uses common, everyday language. |
| 4 | Medical terms are used only to familiarize audience with the terms. When used, medical terms are defined. |
| 5 | The material uses the active voice. |
| **Use of Numbers** | |
| 6 | Numbers appearing in the material are clear and easy to understand. |
| 7 | The material does not expect the user to perform calculations. |
| **Organization** | |
| 8 | The material breaks or “chunks” information into short sections. |
| 9 | The material’s sections have informative headers. |
| 10 | The material presents information in a logical sequence. |
| 11 | The material provides a summary. |
| **Layout & Design** | |
| 12 | The material uses visual cues (e.g., arrows, boxes, bullets, bold, larger font, highlighting) to draw attention to key points. |
| **Use of Visual Aids** | |
| 15 | The material uses visual aids whenever they could make content more easily understood (e.g., illustration of healthy portion size). |
| 16 | The material’s visual aids reinforce rather than distract from the content. |
| 17 | The material’s visual aids have clear titles or captions. |
| 18 | The material uses illustrations and photographs that are clear and uncluttered. |
| 19 | The material uses simple tables with short and clear row and column headings. |
| **Actionability** | |
| 20 | The material clearly identifies at least one action the user can take. |
| 21 | The material addresses the user directly when describing actions. |
| 22 | The material breaks down any action into manageable, explicit steps. |
| 23 | The material provides a tangible tool (e.g., menu planners, checklists) whenever it could help the user take action. |
| 24 | The material provides simple instructions or examples of how to perform calculations. |
| 25 | The material explain show to use the charts, graphs, tables, or diagrams to take actions. |
| 26 | The material uses visual aids whenever they could make it easier to act on the instructions. |

**Supplemental table 4: Inter-Rater Consistency of Evaluation Metrics Across Pediatric Reviewers**

| Variables  M (Q₁, Q₃) | YZ | YY | HJC | TX | Total (n = 400) | χ²# | P |
| --- | --- | --- | --- | --- | --- | --- | --- |
|  |  |  |  |  |  |  |  |
| DISCERN Section A | 17  (15·75,24) | 18·50 (16,26) | 19·5 (16·75,27·25) | 18·50 (15,25·25) | 18  (16, 26) | 6·22 | 0·10 |
| DISCERN Section B | 10 (7,14) | 10 (7,14) | 9·5 (7,15) | 9 (7,15) | 10 (7, 14) | 0·62 | 0·89 |
| DISCERN Section C | 3 (3,3) | 3 (3,3) | 3 (3,3) | 3 (3,3) | 3 (3, 3) | 3·93 | 0·27 |
| DISCERN Total score | 33 (28,39·25) | 34 (29,40) | 36 (29,42) | 34 (27,41) | 34 (28, 41) | 3·81 | 0·28 |
| Likert Scales Section A:Accuracy | 4 (4,4) | 4 (4,4) | 4 (4,5) | 4 (4,5) | 4 (4, 5) | 7·91 | 0·05 |
| Likert Scales Section B:Completeness | 3 (2,4) | 3 (2,4) | 3 (3,4) | 3 (3,4) | 3 (2, 4) | 4·46 | 0·22 |
| Likert Scales Section C:Comprehensibility | 4 (4,5) | 4 (4,5) | 4 (4,5) | 4 (4,5) | 4 (4, 5) | 5·71 | 0·13 |
| Likert Scales  Total score | 11 (10,12) | 11 (10,12) | 12 (11,13) | 12 (10,13) | 12 (10, 13) | 7·39 | 0·06 |
| PEMAT-P Understandability | 0·75 (0·67,0·84) | 0·77 (0·67,0·83) | 0·77 (0·69,0·85) | 0·77 (0·67,0·84) | 0·77  (0·67, 0·85) | 4·62 | 0·20 |
| PEMAT-P Actionability | 0 (0,0·23) | 0 (0,0·2) | 0 (0,0·2) | 0·2 (0,0·35) | 0 (0, 0·2) | 4·39 | 0·22 |

# Kruskal-waills test

M: Median, Q₁: 1st Quartile, Q₃: 3st Quartile
